# Diabetes epidemiology: Analysis of trends over time

**DOI:** 10.1111/1753-0407.13341

**Published:** 2022-12-09

**Authors:** Zachary T. Bloomgarden

**Affiliations:** ^1^ Department of Medicine, Division of Endocrinology, Diabetes and Bone Disease Icahn School of Medicine at Mount Sinai New York New York USA

A useful analysis approach is to look over time at changes in relationships between various conditions and outcomes. A fascinating graphic website based on data from the Global Burden of Disease study allows comparison of deaths, years of life lost, and various measures of disability from various causes over time from 1990 to 2019 (https://vizhub.healthdata.org/gbd-compare/). Using this tool, diabetes accounted for 1.42% of global deaths in 1990 and for 2.74% in 2019. the tool allows estimation of diabetes deaths from metabolic, behavioral, and environmental causes; of deaths from type 1 diabetes (T1D) and type 2 diabetes (T2D); of deaths from chronic kidney disease; and many other potential variables, and I encourage the reader to visit the site and experiment! Several recent articles exemplify the study of changes over time, giving fascinating insight into various aspects of diabetes and its complications.

A group of investigators reported a T1D global microsimulation model, estimating that the incidence rate of T1D increased from around 9 to 13.7 per 100 000 persons from 1990 to 2021, with a projected further 1.29‐fold increase to 17.6 per 100 000 in 2050. Incidence varied substantially by region, from 3.4 per 100 000 in Japan to 61.9 per 100 000 in Finland. In addition, the investigators found marked regional variability in the prevalence of undiagnosed T1D, ranging from 5% in Australia and New Zealand, North America, and western and northern Europe, to more than 65% in west Africa, south and southeast Asia, and Melanesia.^1^

Using data from high‐income regions with >1.5 billion person‐years of observation, with >5 million persons developing T2D between 2005 and 2019, the lifetime risk of diabetes before age 80 ranged from 14% to 54%. Despite the decrease over time in association with a decrease in T2D incidence in most of the regions studied, lifetime risk generally exceeded 30%. Life expectancy for persons with T2D at age 20 was 44–59 years in men and 54–64 years in women, and T2D was associated with a range from 3 to 13 years of life lost, particularly with development at younger ages.^2^

A study using administrative health care data for >10 000 000 persons from Ontario, Canada analyzed the 5‐year risk association between diabetes and cardiovascular disease (CVD) from 1994 to 2019, during which time the prevalence of diabetes increased from 3.1% to 9.0% and that of CVD increased from 2.5% to 3.7% of the population. The relative risk of CV events with either diabetes alone or CVD alone in 1994 was 2.06‐ and 2.16‐fold greater than in persons with neither condition. In 2014 the relative risk of a CV event in persons with diabetes was 1.58‐fold, whereas that of persons with CVD alone was 2.06‐fold greater than in those with neither condition, leading the authors to suggest that “diabetes is still an important cardiovascular risk factor but no longer equivalent to CVD”^3^

The German/Austrian Diabetes Patient Follow‐up Registry (Diabetes‐Patienten‐Verlaufsdokumentation—DPV), the England/Wales National Pediatric Diabetes Audit, and the Type 1 Diabetes Exchange in the United States reported trends among persons with T1D averaging age 13.5; insulin pumps were used by 41%, 19%, and 56% of those in the respective groups in 2012, and by 55%, 41%, and 73% in 2018, and continuous glucose monitoring use increased from 4% to 6% in 2010 to 64%, 13%, and 40% in the respective groups in 2018. However, glycosylated hemoglobin (HbA1c) averaged 7.7%, 8.7%, and 8.0% in 2010 and 7.6%, 7.9%, and 8.5% in 2018, leading the authors to suggest that simple adoption of pump and continuous glucose monitor use may be “insufficient to reduce HbA1c”.^4^
